# Predictive accuracy of partial coherence interferometry and swept-source optical coherence tomography for intraocular lens power calculation

**DOI:** 10.1038/s41598-018-32246-z

**Published:** 2018-09-13

**Authors:** Woong-Joo Whang, Young-Sik Yoo, Min-Ji Kang, Choun-Ki Joo

**Affiliations:** 1 Department of Ophthalmology and Visual Science, Yeouido St. Mary’s Hospital, College of Medicine, The Catholic University of Korea, Seoul, Korea; 2 Department of Ophthalmology and Visual Science, Seoul St. Mary’s Hospital, College of Medicine, The Catholic University of Korea, Seoul, Korea

## Abstract

The purpose of this study is to compare the predictive accuracy of intraocular lens (IOL) calculations made with partial coherence interferometry (PCI, IOLMaster, version 5) and swept-source optical coherence tomography (SS-OCT, Argos). Axial length (AL), mean keratometry value (K), and anterior chamber depth (ACD) were obtained using PCI and SS-OCT optical biometers. Intraocular lens (IOL) power calculations were made using the Barret-Universal II, Haigis, Hoffer Q, SRK/T, and T2 formulas and compared the predictive accuracy between biometers. In 153 eyes (153 patients), axial length measurements made with PCI (24.65 ± 2.35 mm) and SS-OCT (24.62 ± 2.29 mm) were significantly different (P < 0.001). Corneal power (P = 0.97) and anterior chamber depth (P = 0.51) were not significantly different between biometer. The mean absolute error was not significantly different between the five IOL power calculation formulas for either PCI or SS-OCT measurements. When AL was 24.5–26.0 mm, mean absolute error derived from SS-OCT was smaller than mean absolute error derived from PCI for all five IOL power calculation formulas (all P < 0.05). In conclusion, predictive accuracy of PCI and SS-OCT were nearly the same. However, in medium-long eyes, the predictive accuracy of SS-OCT for IOL calculations was higher.

## Introduction

Optical biometry measurements made with partial coherence interferometry (PCI) produce better intraocular lens (IOL) power predictions and refractive outcomes following cataract surgery than ultrasound biometry measurements^1–11^. Swept-source optical coherence tomography (SS-OCT) with 1050–1060 nm light has also been used to obtain biometric measurements because of higher source light penetration than prior biometers that used 780–840 nm light^12^. Some SS-OCT optical biometers have been introduced for clinical use, including the OA-2000 (Tomey Corp.), IOLMaster 700 (Carl Zeiss Meditec, AG), and Argos (Movu, Inc.). Axial length (AL) measurements made with SS-OCT (Argos, Movu, Inc.) were comparable to those obtained with older generation biometers, including PCI. Additionally, SS-OCT biometric measurements were repeatable and reproducible^12^.

Slight differences in biometric measurements (e.g., AL, corneal power, and anterior chamber depth [ACD]) between measurement modalities require that IOL constants be optimized for each instrument^13^. Prior studies have shown that OA-2000 had a higher predictive accuracy than PCI measurements^14^, but that the IOLMaster 700 and PCI produced equally predicable IOL power calculations^15^. However, no prior studies have investigated the predictive accuracy of Argos biometer. The current study retrospectively examines SS-OCT (Argos) and PCI (IOLMaster) IOL power calculation predictive accuracy made with five commonly used IOL power calculation formulas.

## Materials and Methods

The Institutional Review Board for Human studies at Seoul St. Mary Hospital (Seoul, Korea) reviewed and approved this study protocol. Informed consent was obtained from all patients before beginning data collection and analyses. All study conduct adhered to the tenets of the Declaration of Helsinki for the use of human participants in biomedical research.

### Patients

This retrospective study included patients who underwent uncomplicated conventional cataract surgery between May and November of 2016. Only one eye of each patient was included in analyses. Patients were excluded if any of the following were true: history of ocular surgery, postoperative best-corrected distance visual acuity worse than 20/40, or the presence of corneal disease, pseudoexfoliation, zonular weakness, large corneal astigmatism (>3.00 D), glaucoma, macular disease, or amblyopia.

### Optical biometers

Preoperative IOL power calculations were performed using both PCI (IOLMaster, version 5, Carl Zeiss Meditec, Inc., Germany) and SS-OCT (Argos, Movu, Inc., Japan) biometers. Both instruments measure AL as the distance from the anterior corneal apex to the retinal pigment epithelium. PCI uses an infrared laser (780 nm) to measure AL. It projects 6 light spots arranged in a circle onto the cornea (projected radius of 2.3–2.5 mm). Spot reflections are recorded and the distances separating opposite spots are measured. These distances are then used to calculate toroidal surface curvature. The lateral slit illumination technique is used to measure anterior chamber depth (ACD), which is defined as the distance (along the visual axis) between the corneal epithelium and the anterior lens surface. The Argos uses a 1060 ± 10 nm wavelength swept-source to collect cross-sectional images of the entire eye. The system uses three OCT images to measure AL and ACD for every calculation. Keratometry is performed using a 2.1 mm diameter ring made up of 16 infrared light emitting diodes (LEDs).

### Cataract surgery

All cataract surgeries were performed by one surgeon (CKJ) through a 2.2 mm clear corneal incision and a continuous curvilinear capsulorhexis (CCC). Surgeries were performed under local anaesthesia (topical 4% lidocaine, 0.5% proparacaine hydrochloride; Alcaine, Alcon Laboratories, Fort Worth, TX) and phacoemulsification was performed using the Ozil torsional hand piece with the Infiniti Vision System (Alcon Laboratories) set at 100% torsional ultrasound, 350 mm Hg vacuum, and 35 cc/min aspiration. Following phacoemulsification, all patients underwent IOL (Precizon monofocal 560, Ophtec, Groningen, Netherland) insertion into the capsular bag. No intraoperative complications occurred in included patients.

### Visual and refractive outcomes

Refractive outcomes were measured 3 months after surgery using manual refraction. Predicted refraction was assessed using Barret-Universal II, Haigis, Hoffer Q, SRK/T, and T2 formulas (Electronic supplementary material). Optimization was performed by applying optimized IOL constants, published by the User Group for Laser Interference Biometry (ULIB) (http://www.augenklinik.uni-wuerzburg.de/ulib/c1.htm), and was based on PCI biometry measurements. Refractive outcomes were determined using The Barret-Universal II, Hoffer Q, SRK/T, and T2 formulas and retrospectively personalized by adjusting the IOL constant to produce a mean error of zero. Haigis formula IOL constants (a0, a1, and a2) were determined using linear regression analysis using retrospectively calculated effective lens position (ELP) and the following thin-lens formula:

$${\rm{IOL}}\,{\rm{power}}=\frac{1336}{{\rm{AL}}-{\rm{ELP}}}-\frac{1336}{\frac{1336}{Z}\,-\,{\rm{ELP}}}$$

$${\rm{Z}}=\frac{({\rm{nc}}-1)1000}{{\rm{r}}}+\frac{1000}{\frac{1000}{PostRx}-{\rm{VD}}}$$

where, nc is the keratometric index of refraction, r is the corneal radius, PostRx is the postoperative refraction, and VD is the vertex distance.

Personalization was performed using Microsoft Excel (Redmond, WA). Optimized and personalized IOL constants are listed in Table 1.

**Table 1:** Optimized IOL constants and personalized IOL constants for the PCI and SS-OCT.

	Optimized constants	Personalized constants	
		PCI	SS-OCT
Barret-Universal II	Lens factor = 1.62	Lens factor = 1.25	Lens factor = 1.21
Haigis	a0 = 1.020	a0 = −0.229	a0 = 1.190
	a1 = 0.400	a1 = 0.195	a1 = 0.317
	a2 = 0.100	a2 = 0.178	a2 = 0.100
Hoffer Q	pACD = 5.26	pACD = 5.11	pACD = 5.05
SRK/T	A = 118.50	A = 118.08	A = 118.01
T2	A = 118.50	A = 118.08	A = 117.99

Prediction error was defined as the actual postoperative spherical equivalent (SE) minus the predicted SE and mean error (ME) was mean value of prediction error. Mean absolute error (MAE) and median absolute error (MedAE) represented the mean and median values of the absolute value of prediction error. We also calculated the percentage of eyes with an ME within ± 0.25, ± 0.50, and ± 1.00 D.

Included patients were classified into the following subgroups according to the average AL value of both biometer measurements: short eyes (AL < 22.0 mm), medium eyes (22.0 mm ≤ AL < 24.5 mm), medium-long eyes (24.5 mm ≤ AL < 26.0 mm), and long eyes (AL ≥ 26.0 mm). The ME, MAE, and MedAE were examined in each subgroup when personalized IOL constants were used.

### Data analyses

Mean corneal power, AL, ACD, ME, and MAE were compared between groups using Wilcoxon-signed rank tests. Pearson’s correlation tests were used to evaluate correlations between optical biometers. Measurements between biometers were said to agree if they were within the mean ± 2 standard deviations of the difference between biometer measurements (PCI-SS-OCT). Chi-square tests were also performed to compare the percentage of eyes with an ME within ±0.25, ±0.50, and ±1.00 D. Statistical analyses were performed using SPSS statistical software (version 19.0, SPSS, Inc., USA) and statistical significance was defined as P < 0.05.

## Results

**Figure 1:**
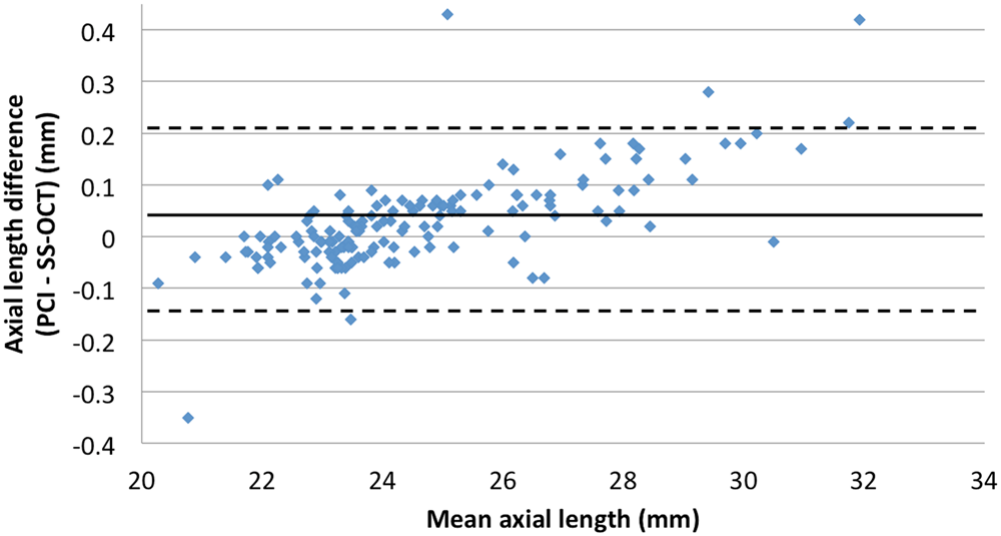
Bland-Altman plot for the axial length measurement. (PCI = partial coherence interferometry; SS-OCT = swept-source optical coherence tomography) The limits of agreement were set at ±1.96 × standard deviation (SD).

**Figure 2:**
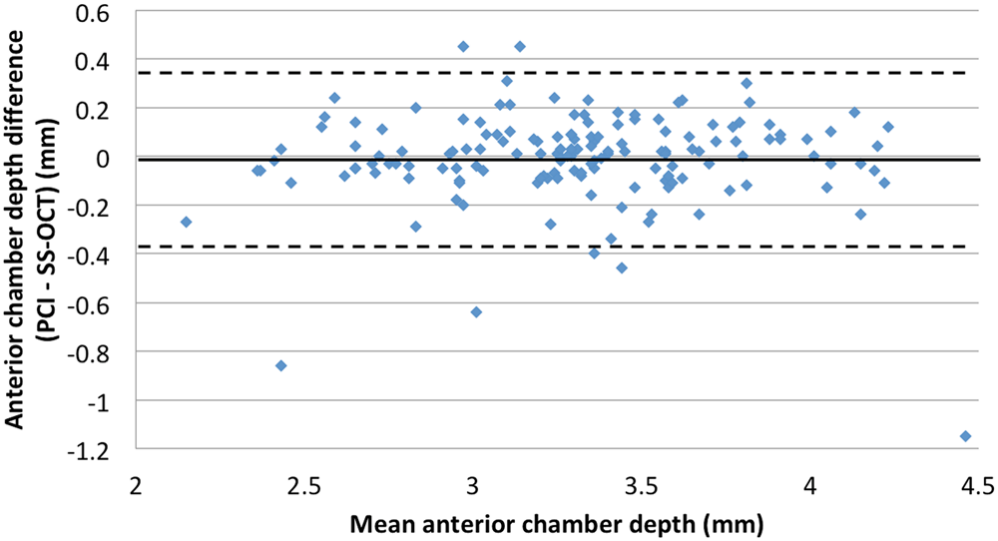
Bland-Altman plot for the anterior chamber depth measurement. (PCI = partial coherence interferometry; SS-OCT = swept-source optical coherence tomography) The limits of agreement were set at ±1.96 × standard deviation (SD).

**Figure 3:**
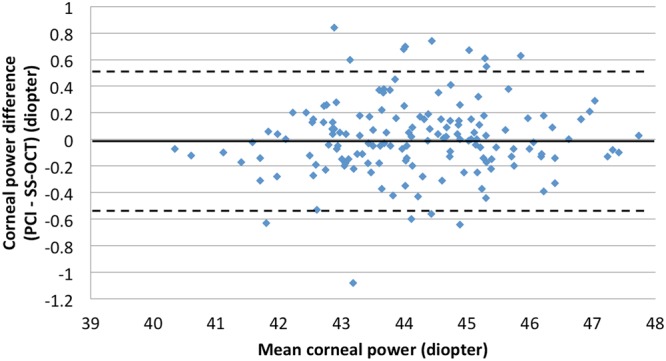
Bland-Altman plot for the mean corneal power measurement. (PCI = partial coherence interferometry; SS-OCT = swept-source optical coherence tomography) The limits of agreement were set at ±1.96 × standard deviation (SD).

A total of 153 eyes (75 left, 78 right) of 153 patients (83 women, 70 men) were included in analyses. Mean subject age was 64.84 ± 8.76 years (range: 47–81 years) and mean cataract grade was 2.53 ± 1.13. Patient demographic and ocular characteristics are summarized in Table 2. Axial length measurements made with SS-OCT (24.62 ± 2.29 mm) were significantly shorter than those made with PCI (24.65 ± 2.35 mm, *P* value < 0.001). However, the agreement and correlation between AL measurement types were good (*r* = 1.000, *P* < 0.001; Fig. 1). There was no significant difference between SS-OCT and PCI measurements in ACD (P = 0.51,) or mean corneal power (*P* = 0.97). Additionally, both measurement methods were in good agreement and were highly correlated (ACD: *r* = 0.914, *P* < 0.001, Fig. 2; mean corneal power: *r* = 0.981, *P* < 0.001, Fig. 3).

**Table 2:** Patient characteristics and biometric data (axial length, anterior chamber depth, and corneal power) by PCI and SS-OCT.

	PCI	SS-OCT	^†^*P* value
Eyes	153		
**Preoperative**			
Cataract grade (Emery-Little)	2.53 ± 1.13 (grade 1: 35 eyes; grade 2: 36 eyes; grade 3: 57 eyes; grade 4: 16 eyes; grade 5: 9 eyes)		
UDVA (logMAR)	0.59 ± 0.42		
CDVA (logMAR)	0.43 ± 0.45		
Spherical equivalent (diopter)	−1.62 ± 4.75		
Age	64.84 ± 8.76		
Axial length (mm)	24.65 ± 2.35	24.62 ± 2.29	<0.001
Anterior chamber depth (mm)	3.30 ± 0.46	3.31 ± 0.46	0.51
Mean corneal power (diopter)	44.19 ± 1.47	44.18 ± 1.45	0.97
IOL power (diopter)	18.07 ± 5.43		
**Postoperative**			
UDVA (logMAR)	0.16 ± 0.20		
CDVA (logMAR)	0.02 ± 0.06		
Spherical equivalent (diopter)	−1.20 ± 1.03		

^†^Wilcoxon signed rank test.

Abbreviations: UDVA = uncorrected distance visual acuity; CDVA = corrected distance visual acuity; IOL = intraocular lens.

Refractive outcomes made with SS-OCT and PCI biometric parameter measurements using optimized IOL constants are shown in Table 3. Using these optimized IOL constants would have led to postoperative myopia, which would have been more severe with SS-OCT measurements and the Haigis (PCI: −0.07 ± 0.45 D, SS-OCT: −0.13 ± 0.44 D, *P* = 0.045) and SRK/T (PCI: −0.29 ± 0.49 D, SS-OCT: −0.33 ± 0.50 D, *P* = 0.034) formulas. Predicted refraction results after IOL constants personalization are shown for each IOL power calculation equation in Table 4. Both MAE and MedAE were lower after IOL constants personalization than before personalization. There were no significant differences between SS-OCT and PCI biometry measurements.

**Table 3:** Mean (arithmetic) error, mean absolute error, median absolute error percentage of eyes with an error of prediction of ±0.25, ±0.50 and ±1.00 diopter and range of prediction error when the optimized IOL constants were applied.

		PCI	SS-OCT	^†^*P* value
Barret-Universal II	ME (D)	−0.38 ± 0.44	−0.43 ± 0.45	0.063
	MAE (D)	0.49 ± 0.33	0.51 ± 0.35	0.53
	MedAE (D)	0.44	0.50	
	±0.25 D (%)	26.8	28.1	0.90
	±0.50 D (%)	57.5	51.6	0.36
	±1.00 D (%)	91.5	92.2	1.00
	Range (D)	−1.70~0.93	−1.74~0.86	
Haigis	ME (D)	−0.07 ± 0.45	−0.13 ± 0.44	0.045
	MAE (D)	0.36 ± 0.28	0.36 ± 0.28	0.82
	MedAE (D)	0.29	0.30	
	±0.25 D (%)	45.1	43.8	0.91
	±0.50 D (%)	73.9	73.2	1.00
	±1.00 D (%)	96.1	96.7	1.00
	Range (D)	−1.28~1.21	−1.30~1.21	
Hoffer Q	ME (D)	−0.16 ± 0.48	−0.20 ± 0.46	0.069
	MAE (D)	0.41 ± 0.28	0.42 ± 0.28	0.87
	MedAE (D)	0.40	0.39	
	±0.25 D (%)	37.3	34.0	0.63
	±0.50 D (%)	65.4	66.7	0.90
	±1.00 D (%)	96.1	96.7	1.00
	Range	−1.24~1.29	−1.24~1.10	
SRK/T	ME (D)	−0.29 ± 0.49	−0.33 ± 0.50	0.034
	MAE (D)	0.47 ± 0.33	0.49 ± 0.34	0.15
	MedAE (D)	0.41	0.46	
	±0.25 D (%)	28.8	26.8	0.80
	±0.50 D (%)	64.1	56.2	0.20
	±1.00 D (%)	91.5	91.5	1.00
	Range (D)	−1.50~1.40	−1.64~1.54	
T2	ME (D)	−0.29 ± 0.47	−0.33 ± 0.48	0.086
	MAE (D)	0.45 ± 0.32	0.48 ± 0.33	0.09
	MedAE (D)	0.41	0.46	
	±0.25 D (%)	34.6	27.5	0.22
	±0.50 D (%)	63.4	57.5	0.35
	±1.00 D (%)	94.8	93.5	0.81
	Range (D)	−1.46~1.31	−1.67~1.46	

^†^Wilcoxon signed rank test for ME,MAE, MedAE and Fisher’s exact test for the percentages of ±0.25 D, ±0.50 D, ±1.00 D (%).

Abbreviations: D = diopter; ME = mean error; MAE = mean absolute error; MedAE = median absolute error.

**Table 4:** Mean (arithmetic) error, mean absolute error, median absolute error, percentage of eyes with an error of prediction of ±0.25, ±0.50 and ±1.00 diopter and range of prediction error when the retrospectively personalized IOL constants were applied.

		PCI	SS-OCT	^†^*P* value
Barret-Universal II	ME (D)	0.00 ± 0.44	0.00 ± 0.45	0.83
	MAE (D)	0.34 ± 0.29	0.36 ± 0.27	0.54
	MedAE (D)	0.27	0.32	
	±0.25 D (%)	49.0	41.2	0.21
	±0.50 D (%)	75.2	75.8	1.00
	±1.00 D (%)	96.7	97.4	1.00
	Range (D)	−1.32~1.31	−1.31~1.28	
Haigis	ME (D)	0.00 ± 0.45	0.00 ± 0.44	0.34
	MAE (D)	0.35 ± 0.29	0.34 ± 0.27	0.68
	MedAE (D)	0.28	0.30	
	±0.25 D (%)	47.7	46.4	0.91
	±0.50 D (%)	73.2	75.2	0.79
	±1.00 D (%)	96.7	96.7	1.00
	Range (D)	−1.26~1.29	−1.24~1.26	
Hoffer Q	ME (D)	0.00 ± 0.48	0.00 ± 0.46	0.67
	MAE (D)	0.39 ± 0.27	0.37 ± 0.27	0.24
	MedAE (D)	0.35	0.35	
	±0.25 D (%)	35.3	41.2	0.35
	±0.50 D (%)	69.9	71.9	0.80
	±1.00 D (%)	98.0	98.0	1.00
	Range (D)	−1.08~1.45	−1.03~1.31	
SRK/T	ME (D)	0.00 ± 0.49	0.00 ± 0.50	0.53
	MAE (D)	0.38 ± 0.31	0.39 ± 0.31	0.79
	MedAE (D)	0.31	0.33	
	±0.25 D (%)	45.1	42.5	0.73
	±0.50 D (%)	69.3	68.6	1.00
	±1.00 D (%)	97.4	96.7	1.00
	Range (D)	−1.21~1.69	−1.30~1.87	
T2	ME (D)	0.00 ± 0.47	0.00 ± 0.48	0.70
	MAE (D)	0.37 ± 0.28	0.38 ± 0.29	0.90
	MedAE (D)	0.32	0.34	
	±0.25 D (%)	43.1	39.2	0.64
	±0.50 D (%)	73.9	73.2	1.00
	±1.00 D (%)	96.7	96.7	1.00
	Range (D)	−1.17~1.60	−1.34~1.79	

^†^Wilcoxon signed rank test for ME,MAE, MedAE and Fisher’s exact test for the percentages of ±0.25 D, ±0.50 D, ±1.00 D (%).

Abbreviations: D = diopter; ME = mean error; MAE = mean absolute error; MedAE = median absolute error.

Eyes were divided into preoperative AL groups as described above. Table 5 summarizes demographic and ocular characteristics of patients with short (11 eyes), medium (80 eyes), medium-long (23 eyes), and long (39 eyes) eyes. In short eyes, SS-OCT AL measurements (21.51 ± 0.56 mm) were significantly greater than PCI AL measurements (21.44 ± 0.61 mm, *P* value = 0.01) The SS-OCT ACD measurements (2.75 ± 0.26 mm) were also significantly larger than PCI ACD measurements (2.61 ± 0.37 mm, *P* = 0.04). In contrast, SS-OCT AL measurements were smaller than PCI AL measurements in both medium-long (SS-OCT: 25.05 ± 0.38 mm, PCI: 25.12 ± 0.40 mm, *P* < 0.001) and long (SS-OCT: 27.91 ± 1.57 mm, PCI: 28.02 ± 1.63 mm, *P* < 0.001) eyes. All other measurements were comparable between SS-OCT and PCI in all AL subgroups.

**Table 5:** Patient characteristics and biometric data (axial length, anterior chamber depth, and corneal power) by PCI and SS-OCT in 4 groups classified according to axial length.

		PCI	SS-OCT	^†^*P* value
Short (n = 11)	Axial length (mm)	21.44 ± 0.61	21.51 ± 0.56	0.01
	Anterior chamber depth (mm)	2.61 ± 0.37	2.75 ± 0.26	0.04
	Mean corneal power (diopter)	46.47 ± 1.31	46.42 ± 1.36	0.18
Medium (n = 80)	Axial length (mm)	23.32 ± 0.61	23.32 ± 0.60	0.23
	Anterior chamber depth (mm)	3.13 ± 0.34	3.14 ± 0.37	0.79
	Mean corneal power (diopter)	44.37 ± 1.17	44.34 ± 1.18	0.55
Medium-long (n = 23)	Axial length (mm)	25.12 ± 0.40	25.05 ± 0.38	<0.001
	Anterior chamber depth (mm)	3.51 ± 0.32	3.51 ± 0.46	0.25
	Mean corneal power (diopter)	43.40 ± 1.62	43.39 ± 1.52	0.63
Long (n = 39)	Axial length (mm)	28.02 ± 1.63	27.91 ± 1.57	<0.001
	Anterior chamber depth (mm)	3.71 ± 0.33	3.67 ± 0.33	0.08
	Mean corneal power (diopter)	43.63 ± 1.23	43.69 ± 1.23	0.17

^†^Wilcoxon signed rank test.

Table 6 summarizes ME, MAE, and MedAE of each AL subgroup when personalized IOL constants were applied. In short eyes, ME was significantly different between SS-OCT and PCI IOL power calculations when the Barret-Universal II, Hoffer Q, and SRK/T formulas were used. Specifically, PCI had more myopic results for the Hoffer Q formula and SS-OCT had more hyperopic results for the Barret-Universal II and SRK/T formulas. In medium and long eyes, there were no significant differences in ME, MAE or MedAE between SS-COT and PCI calculations. However, in medium-long eyes, SS-OCT calculations had a significantly smaller MAE than PCI calculations, except when the SRK/T formula was used. Additionally, MedAEs were smaller in medium-long eyes for all formulas examined when SS-OCT was used to calculate IOL power.

**Table 6:** Mean (arithmetic) error, mean absolute error, and median absolute error in 4 groups classified according to axial length when the retrospectively personalized IOL constants were applied.

Range	Formula		PCI	SS-OCT	^†^*P* value
Short	Barret-Universal II	ME (D)	0.06 ± 0.41	0.27 ± 0.50	0.04
		MAE (D)	0.35 ± 0.21	0.43 ± 0.36	0.72
		MedAE (D)	0.28	0.30	
	Haigis	ME (D)	0.25 ± 0.51	0.21 ± 0.52	0.37
		MAE (D)	0.46 ± 0.32	0.43 ± 0.34	0.86
		MedAE (D)	0.40	0.40	
	Hoffer Q	ME (D)	−0.21 ± 0.41	0.01 ± 0.51	0.03
		MAE (D)	0.38 ± 0.25	0.42 ± 0.26	0.48
		MedAE (D)	0.42	0.38	
	SRK/T	ME (D)	0.09 ± 0.71	0.29 ± 0.81	0.02
		MAE (D)	0.52 ± 0.47	0.61 ± 0.59	0.93
		MedAE (D)	0.43	0.34	
	T2	ME (D)	0.10 ± 0.65	0.29 ± 0.75	0.70
		MAE (D)	0.47 ± 0.44	0.57 ± 0.55	0.90
		MedAE (D)	0.37	0.29	
Medium	Barret-Universal II	ME (D)	0.04 ± 0.46	0.06 ± 0.44	0.25
		MAE (D)	0.35 ± 0.30	0.37 ± 0.25	0.32
		MedAE (D)	0.27	0.33	
	Haigis	ME (D)	−0.02 ± 0.45	−0.01 ± 0.44	0.57
		MAE (D)	0.35 ± 0.29	0.36 ± 0.25	0.54
		MedAE (D)	0.25	0.33	
	Hoffer Q	ME (D)	−0.09 ± 0.46	−0.05 ± 0.46	0.16
		MAE (D)	0.38 ± 0.27	0.39 ± 0.25	0.77
		MedAE (D)	0.34	0.38	
	SRK/T	ME (D)	−0.01 ± 0.48	0.04 ± 0.46	0.06
		MAE (D)	0.38 ± 0.31	0.39 ± 0.31	0.72
		MedAE (D)	0.27	0.35	
	T2	ME (D)	0.03 ± 0.46	0.06 ± 0.45	0.15
		MAE (D)	0.37 ± 0.27	0.39 ± 0.24	0.60
		MedAE (D)	0.26	0.35	
Medium-long	Barret-Universal II	ME (D)	0.09 ± 0.44	0.00 ± 0.29	0.20
		MAE (D)	0.34 ± 0.29	0.21 ± 0.19	0.017
		MedAE (D)	0.25	0.13	
	Haigis	ME (D)	−0.01 ± 0.44	−0.04 ± 0.28	0.98
		MAE (D)	0.34 ± 0.26	0.21 ± 0.18	0.006
		MedAE (D)	0.26	0.19	
	Hoffer Q	ME (D)	0.05 ± 0.46	−0.04 ± 0.27	0.14
		MAE (D)	0.36 ± 0.28	0.21 ± 0.16	0.003
		MedAE (D)	0.35	0.19	
	SRK/T	ME (D)	0.04 ± 0.50	−0.05 ± 0.44	0.10
		MAE (D)	0.34 ± 0.37	0.29 ± 0.33	0.68
		MedAE (D)	0.21	0.21	
	T2	ME (D)	0.06 ± 0.44	−0.04 ± 0.32	0.14
		MAE (D)	0.34 ± 0.29	0.24 ± 0.21	0.007
		MedAE (D)	0.32	0.17	
Long	Barret-Universal II	ME (D)	−0.14 ± 0.41	−0.20 ± 0.45	0.14
		MAE (D)	0.32 ± 0.29	0.39 ± 0.30	0.18
		MedAE (D)	0.25	0.38	
	Haigis	ME (D)	−0.11 ± 0.42	−0.06 ± 0.47	0.22
		MAE (D)	0.33 ± 0.29	0.35 ± 0.31	0.84
		MedAE (D)	0.27	0.21	
	Hoffer Q	ME (D)	0.23 ± 0.47	0.16 ± 0.51	0.17
		MAE (D)	0.43 ± 0.29	0.42 ± 0.33	0.39
		MedAE (D)	0.36	0.39	
Long	SRK/T	ME (D)	−0.03 ± 0.45	−0.10 ± 0.47	0.09
		MAE (D)	0.37 ± 0.24	0.39 ± 0.27	0.82
		MedAE (D)	0.39	0.34	
	T2	ME (D)	−0.12 ± 0.43	−0.19 ± 0.47	0.15
		MAE (D)	0.35 ± 0.27	0.40 ± 0.31	0.26
		MedAE (D)	0.33	0.36	

^†^Wilcoxon signed rank test.

Abbreviations: D = diopter; ME = mean error; MAE = mean absolute error; MedAE = median absolute error.

## Discussion

To the best of our knowledge, this is the first study to evaluate the predictive accuracy of a new SS-OCT system when widely-used IOL power calculation formulas were used. Overall, the predictive accuracies of the two optical biometers were nearly the same when personalized IOL constants were applied. The personalized IOL constants were nearly identical for SS-OCT and PCI biometers, but a slight difference was likely introduced by the different measurement techniques. However, personalized IOL constants derived from both optical biometers differed from the published ULIB optimized IOL constants (http://ocusoft.de/ulib/c1.htm). It should be noted that the ULIB optimized IOL constants were determined using a relatively small sample size and by using single optimized IOL constants with the Haigis formula. Given that the need for optimization is greater for SS-OCT biometry and that optimized constants are based on PCI biometry, this seems acceptable.

Several factors were controlled and protocols for intraocular lens formula accuracy studies (recommended by Hoffer *et al*.^16^) were adhered to precisely compare biometric measurements. Mean errors of SS-OCT and PCI calculations were made to equal zero for each formula. We also analysed both MAE and MedAE. Additionally, only one eye of each patient was included and only one IOL type was implanted in all patients. Postoperative subjective refraction was measured 3 months after surgery and eyes with a best-corrected distance visual acuity worse than 20/40 were excluded.

Our results showed that AL measurements were smaller when measured with SS-OCT than when measured with PCI. All biometers convert optical distance to geometrical distance, with the degree of conversion determined by the medium’s refractive index. The PCI uses a refractive index of 1.3549 across whole eye, while the SS-OCT uses refractive indexes of 1.376 for the cornea, 1.336 for the aqueous and vitreous, and 1.410 for a cataract. It is likely that these applied refractive index differences caused AL measurement differences. In particular, as AL increases, SS-OCT AL measurements become shorter relative to PCI measurements and this result was similar to the study of Higashiyama *et al*.^17^. In contrast, a previous study found no significant difference between IOLMaster 700 and SS-OCT AL measurements when AL measurements were whole area based^18,19^. Additionally, IOLMaster 700 AL measurements have been shown to be greater than PCI AL measurements in myopic eyes^20^. This finding differs from our results that compared IOLMaster and Argos measurements. Further research is needed to compare AL measurements between SS-OCT various instruments and to determine the predictive accuracy of the IOLMaster 700.

Differences between optical biometer AL measurements can affect refractive outcomes. Compared with the PCI, SS-OCT IOL calculations led to postoperative hyperopia in short eyes and postoperative myopia in long eyes. However, when the Haigis formula was used, the opposite occurred and which might be caused by the number of IOL constants. These results may be due to the fact that the current IOL formulas have been retrospectively corrected and evolved based on the postoperative results after ultrasound biometers or PCI biometer were applied. In medium-long eyes, the SS-OCT predictive accuracy was significantly higher than PCI predictive accuracy. This suggests that SS-OCT reproducibility within a specific range is excellent and that appropriately correcting SS-OCT measurements may result in better refractive outcomes, even in short and long eyes.

The current study examined predictability when IOL calculations were performed using two different 3rd generation formulas, which were designed to generate more accurate IOL position predictions by incorporating the effect of corneal curvature^21^. Popular formulas for IOL power calculation, including the SRK/T and Hoffer Q^22^ formulas, are based on thin lens optics, which mathematically replace the cornea and lens (crystalline or IOL) with infinitely thin lenses of two different refractive powers. Second generation formulas have been retrospectively calibrated after determining the relationship between preoperative AL (measured by ultrasound biometry) and postoperative refractive outcomes. This information was used to create 3^rd^ generation formulas, including the Hoffer Q and SRK/T formulas. However, Sheard *et al*.^23^ found a systematic error in corneal height prediction by the SRK/T formula. They created the T2 formula, a modified SRK/T equation that used a regression formula derived from a large patient sample to correct for corneal height prediction errors. They concluded that the T2 formula can serve as a direct substitute for the SRK/T formula and recommended that the same A-constant be used. In agreement, we found nearly the same A-constant value in the current study after retrospective personalization. The Haigis formula differs from the SRK/T formula in that it uses preoperative ACD instead of preoperative corneal power^4^. This formula was introduced at nearly the same time as the IOLMaster, which is commonly used around the world. The Barret-Universal II formula is the newest formula and has been shown to be most accurate when PCI biometry is used^24,25^. The current study found that the Barret-Universal II formula was more appropriate for PCI biometry in both long (AL ≥ 26 mm) and short (AL < 22 mm) eyes. The Barret-Universal II formula provided a result close to the emmetropic target and had a smaller MAE and MedAE for PCI biometry than for SS-OCT biometry in long and short eyes. The Barret-Universal II formula was designed by analysing optical biometer (e.g., PCI and optical low-coherence reflectometry) measurements and post-operative results. Therefore, applying retrospectively optimized characteristics that were optimized by a PCI biometer may have influenced our study results.

Our study had several limitations. First, only eyes in which biometry was successfully performed with two optical biometers were included. Therefore, success rates of acquiring AL measurements were not examined. The SS-OCT uses a longer wavelength (1060 nm) than PCI (780 nm). Therefore, SS-OCT can image deeper than PCI and, potentially, across a cataract. Shammas *et al*.^12^ reported that AL was successfully measured in 96% and 77% of eyes with a cataract using SS-OCT and PCI, respectively. Therefore, our study population generally had less severe cataracts, which may have hindered precise biometer comparison. Second, lens thickness was not factored into IOL power calculations. The Barret-Universal II needs optional biometric measurements to predict IOL power. Because PCI does measure lens thickness, target refractions were calculated using only essential biometry measurements. Future studies should examine whether or not including optional biometry measures (e.g., lens thickness and corneal diameter) enhance predictive accuracy. Third, we did not perform detailed accuracy comparisons between IOL calculation formulas or sequencing IOL formulas. The primary purpose of this study was to compare predictive accuracy between two optical biometers and not to compare IOL calculation formula accuracy. Selecting appropriate IOL formulas for each AL or other biometric classification will be more reliable in the future after a larger number of cases are analysed. Finally, there was no posterior staphyloma in this study, and only patients with fixation were included. In future studies, it would be necessary to evaluate the predictive accuracy in patients with the above cases.

In conclusion, the predictive accuracies of the Argos SS-OCT and IOLMaster PCI optical biometers are nearly the same, except for in medium-long eyes, in which the predictive accuracy of SS-OCT biometry was higher.

## Electronic supplementary material

Equation for intraocular lens calculation formulas

## Data Availability

The datasets generated during and/or analysed during the current study are available from the corresponding author on reasonable request.
