# Supplementary material for: Predictive accuracy of partial coherence interferometry and swept-source optical coherence tomography for intraocular lens power calculation
Source: Sci Rep. 2018 Sep 13;8:13732. doi: 10.1038/s41598-018-32246-z (PMC6137182; doi:10.1038/s41598-018-32246-z)
Supplement: Supplementary file 1 — Equation for intraocular lens calculation formulas [file 41598_2018_32246_MOESM1_ESM.docx]

**Predictive accuracy of partial coherence interferometry and swept-source optical coherence tomography for intraocular lens power calculation**

Woong-Joo Whang, MD^1^, Young-Sik Yoo^2^, MD, Min-Ji Kang, MD^2^, Choun-Ki Joo, MD, PhD^2^

^1^Department of Ophthalmology and Visual Science, Yeouido St. Mary's Hospital, College of Medicine, The Catholic University of Korea, Seoul, Korea

^2^Department of Ophthalmology and Visual Science, Seoul St. Mary's Hospital, College of Medicine, The Catholic University of Korea, Seoul, Korea

**Corresponding author:** Choun-Ki Joo, MD, PhD

#222 Banpo-daero

Seocho-Gu, Seoul, 137-040, Republic of Korea

Tel: +82-2-2258-7612

Fax: +82-2-533-7405

Email: [ckjoo2@catholic.ac.kr](mailto:ckjoo@catholic.ac.kr)

**Running head:** Predictive accuracy of PCI and SS-OCT

Supplementary Table S1. Intraocular lens calculation formulas.

|  | Haigis | Hoffer Q | SRK/T | T2 |
| --- | --- | --- | --- | --- |
| fc (dipter) | 331.5/rc | 337.5/rc | 333/rc | 333/rc |
| L (mm) | al | al | al + 0.65696 – 0.02029al | al + 0.65696 – 0.02029al |
| IOL constant | a0, a1, a2 | pACD | A constant | A constant |

fc = corneal power; rc = measured anterior corneal radius ; al = measured axial length

**Effective lens position (ELP) for the Haigis formula**

ELP = a0 + a1*acd + a2*al

acd = measured anterior chamber depth

**Effective lens position (ELP) for the Hoffer Q formula**

ELP = pACD + {0.3 * (a-23.5)} + {tan(fc$\frac{\pi}{180}$)^2^ + [0.1*m*(23.5-a)^2^*tan{0.1*(g-a)^2^*$\frac{\pi}{180}$}]

If a > 31 then a = 31;

If a < 18.5 then a = 18.5;

If a $\leq$ 23 then m = 1 else m = -1;

If a $\leq$ 23 then g = 28 else g = 23.5

**Effective lens position (ELP) for the SRK/T formula**

ELP = rc – $\sqrt{{rc}^{2}- \frac{D^{2}}{4}}$ + 0.62467*A constant – 68.747 – 3.336

D = -5.41 + 0.58412*Lcor + $\frac{33.075}{rc}$

If al $\leq$ 24.2 then Lcor = al else Lcor = -3.446 + 1.716*al – 0.0237*al^2^

**Effective lens position (ELP) for the T2 formula**

ELP = -10.326 + 0.32630*al + 0.13533*K + 0.62467*A constant – 68.747 – 3.336

K = corneal power
